# Contextual correlates of happiness in European adults

**DOI:** 10.1371/journal.pone.0190387

**Published:** 2018-01-24

**Authors:** Eva Anna Christina Hart, Jeroen Lakerveld, Martin McKee, Jean-Michel Oppert, Harry Rutter, Hélène Charreire, Ruut Veenhoven, Helga Bárdos, Sofie Compernolle, Ilse De Bourdeaudhuij, Johannes Brug, Joreintje Dingena Mackenbach

**Affiliations:** 1 Department of Epidemiology and Biostatistics, Amsterdam Public Health Research Institute, VU University Medical Center, Amsterdam, the Netherlands; 2 ECOHOST–The Centre for Health and Social Change, London School of Hygiene and Tropical Medicine, London, United Kingdom; 3 Equipe de Recherche en Epidémiologie Nutritionnelle (EREN), Centre de Recherche en Epidémiologie et Statistiques, Inserm (U1153), Inra (U1125), Cnam, COMUE Sorbonne Paris Cité, Université Paris 13, Bobigny, France; 4 Sorbonne Universités, Université Pierre et Marie Curie, Université Paris 06; Institute of Cardiometabolism and Nutrition, Department of Nutrition, Pitié-Salpêtrière Hospital, Assistance Publique-Hôpitaux de Paris, Paris, France; 5 Paris Est University, Lab-Urba, UPEC, Urban School of Paris, Créteil, France; 6 Erasmus Happiness Economics Research Organization, Erasmus Universiteit Rotterdam, Rotterdam, the Netherlands; 7 Optentia Research, North-West University, Vanderbijlpark, South Africa; 8 Department of Preventive Medicine, Faculty of Public Health, University of Debrecen, Debrecen, Hungary; 9 Department of Movement and Sport Sciences, Ghent University, Ghent, Belgium; 10 Amsterdam School of Communication Research (ASCoR), University of Amsterdam, Amsterdam, the Netherlands; Leibniz Institute for Prvention Research and Epidemiology BIPS, GERMANY

## Abstract

**Objectives:**

We aimed to examine the associations of both objectively assessed and perceived physical and social neighborhood characteristics with happiness in European adults. In addition, we aimed to study how these associations differed among subgroups.

**Methods:**

Participants (N = 6037) of the cross-sectional SPOTLIGHT survey reported on their level of happiness using a 5-point Likert scale, and on perceived physical and social environmental neighborhood characteristics. Objective physical environmental characteristics were assessed using a Google Street View-based neighborhood audit. Associations of 14 physical and social environmental characteristics with happiness were analyzed using multivariable multinomial regression analyses with clustered standard errors.

**Results:**

Living in neighborhoods with higher levels of aesthetics and more water and green space was associated with being very happy. Individuals who perceived their neighborhood to be safer, more functional and more aesthetic were more likely to be very happy. The associations of functionality and aesthetics with happiness were strongest in the Ghent region (Belgium), the Randstad (the Netherlands) and Greater London (United Kingdom). Perceived absence of air pollution was only associated with higher levels of happiness in more highly educated participants. Individuals with a larger social network, more social cohesion and who trusted their neighbors were more likely to be very happy. The association between social networks and happiness was somewhat stronger in men than in women. In general, the associations between environmental characteristics and happiness had similar directions and sizes across socio-economic and socio-demographic subgroups.

**Conclusions:**

This European study provided evidence that both objectively assessed and perceived physical and social characteristics of the neighborhood environment are associated with the happiness of its residents.

## Introduction

Happiness is one of the most important values in life [1]. Being happy is associated with healthy lifestyle behaviors and longevity [2–7]. Happiness is a complex concept and can be defined in multiple ways. A commonly used definition of happiness is “the degree to which an individual judges the overall quality of his/her life as a whole favorably” [8–10], in other words, how much one likes the life one lives.

Many studies have identified individual-level factors of happiness, such as biological, personality, lifestyle, socio-demographic and socio-economic factors [5,11–14]. Yet, the contexts or environments in which people live have also been found to be associated with happiness [13,15–18]. Neighborhood environments have increasingly gained attention in epidemiological studies as they contain both physical and social attributes that are likely to be relevant to individual health and well-being [19,20]. This notion is underpinned by the concept of environmental human-friendliness (EHF) [17], which implies that environments may support individuals and groups in pursuing and realizing their goals, which contribute to an higher level of happiness.

Several studies provide evidence for an association between social and physical environmental factors and happiness. For example, social environmental factors such as neighborhood social capital have consistently been positively associated with happiness and related constructs such as subjective well-being [16,21,22]. Some aspects of the physical environment have also been studied extensively; European and American studies found that settings that were aesthetically more attractive and had more green space have consistently been related to higher levels of happiness [15,23–25].

Perceived access to public transport, culture and leisure facilities have been positively linked with happiness in ten major cities in the world (including New York, Paris and Beijing) [15], while another study, in five world cities, found happiness to be more closely correlated with cultural, sporting, shopping and transport amenities (place variables) among young people and with quality of government services (performance variables) among older people [18]. In addition, urbanisation, air pollution and living in a neighborhood of economic disadvantage have been inversely linked to happiness in the US and Europe [26–29]. There is also some evidence from Ireland that proximity to the coast is associated with a higher level of happiness [13]. However, there is a lack of studies investigating multiple social and physical environmental factors in the same sample and across different regions in Europe.

Both the actual environmental characteristics and environmental perceptions may be relevant for happiness [30,31]. However, only a few studies thus far have measured these objective and subjective environmental characteristics at the same time [32,33]. Additionally, some studies have suggested that environment-happiness associations may differ between socio-demographic and socio-economic subgroups, but such evidence is scarce [34]. The aim of this study was thus to explore the physical and social neighborhood environmental correlates of happiness in adults across Europe. We distinguished between perceived and objective measures of the physical environment and explored differences between socio-economic and socio-demographic subgroups.

## Methods

### Study design and study sample

Within the SPOTLIGHT (‘sustainable prevention of obesity through integrated strategies’) project, a cross-sectional study was carried out among inhabitants of urban areas in five different European countries: Ghent and suburbs (Belgium), the Randstad (a conurbation including Amsterdam, Rotterdam and The Hague and Utrecht in the Netherlands), Greater London (UK), Paris and suburbs (France) and Budapest and suburbs (Hungary). Sampling of neighborhoods and recruitment of participants has been described in detail elsewhere [35]. Briefly, neighborhood sampling was based on a combination of residential density and socioeconomic status (SES) data at neighborhood level. This resulted in four types of neighborhoods: low SES/ low residential density, low SES/ high residential density, high SES/ low residential density and high SES/ high residential density. In each urban region, three neighborhoods of each neighborhood type were randomly sampled (i.e. 12 neighborhoods per region, 60 neighborhoods in total).

An online survey among residents of the selected neighborhoods included questions on demographics, neighborhood perceptions, social environmental factors, motivations and barriers for healthy behavior, obesity-related behaviors, weight, height and happiness. A total of 6037 (10.8% response rate) individuals participated in the survey between February and September 2014. The study was approved by the corresponding local ethics committees of participating countries. In Belgium, the study was approved by the ethics committee of Ghent University Hospital, in The Netherlands by the Medical Ethics Committee of the VU University Medical Center in Amsterdam, in Hungary by the Health Science Council, Scientific Research Ethic Committee, in France by the Commission Nationale de l'Informatique et des Libertés and in the UK by The London School of Hygiene & Tropical Medicine Ethics Committee. All participants provided written informed consent.

### Outcome measure–happiness

We assessed level of happiness using a single-item question: ‘In general, how happy are you?’. Measuring happiness by a single item is considered to be reliable, valid, and viable in community surveys as well as in cross-cultural comparisons [36] and our question was similar to questions widely used elsewhere [37]. Our item had five response options (‘very happy’, ‘moderately happy’, ‘no feelings either way’, ‘moderately unhappy’ and ‘very unhappy’). The last two responses were combined as there were very few individuals in the ‘very unhappy’ category (0.8%), which resulted in four categories for analysis.

### Exposure measure–objectively assessed physical environment

The individual level data were supplemented by data from a validated neighborhood audit developed within the SPOTLIGHT project. The methods have been described elsewhere [38]. In brief, characteristics of the physical environment were objectively assessed with the valid and reliable SPOTLIGHT Virtual Audit Tool (S-VAT), which was conducted using Google Street View (GSV) [39]. This tool consists of 41 items on environmental characteristics that were assessed in 4,486 street segments, in 59 neighborhoods (for one Hungarian neighborhood there were no GSV available at the time of the virtual audit) by trained researchers in the SPOTLIGHT project. The characteristics were related to public transport, aesthetics, land use-mix, grocery stores, type of food outlets, recreational, walking and cycling facilities. The proportion of street segments featuring each of the 41 items at neighborhood level was calculated. Further details on these characteristics can be found elsewhere [39,40].

We divided the items into four domains according to Pikora’s framework of environmental determinants: ‘traffic safety’, ‘functionality, ‘destinations’ and ‘aesthetics’[41]. The domain ‘traffic safety’ included characteristics such as the percentage of streets with pedestrian crossings and traffic lights and the domain ‘functionality’ included characteristics such as the percentage of streets with well-maintained sidewalks and traffic calming devices. ‘Destinations’ included characteristics such as the percentage of streets with supermarkets, local shops or restaurants, and ‘aesthetics’ included for example the percentage of streets with well-maintained green areas and good condition residential buildings. A previous study within the same project has demonstrated the reliability of these domains in the current dataset [42]. The domains were treated as continuous variables, with probabilities that a street segment would contain them ranging from 0.01 to 0.71. In addition to the domains, we assessed the percentage of streets alongside bodies of water (e.g. river, canal, lake) and green spaces as a single continuous variable ranging from 0.01 to 1.00.

### Exposure measure–perceived physical environment

We measured the perceived physical environment by asking participants about the characteristics of their neighborhood, using five-point Likert scales. Statements on the neighborhood environment were based on items of the validated ALPHA questionnaire [43] and the Multi Ethnic Study of Atherosclerosis (MESA) instrument [44]. The five response options ranged from ‘strongly disagree’ to ‘strongly agree’. An additional questionnaire item assessed whether or not six specific destination types (e.g. local businesses or facilities) were present in the neighborhood.

Following a similar categorization to the one used for objectively assessed environmental characteristics, four domains in the perceived physical environment were distinguished according the framework of Pikora [41,42]. We assessed the domains as continuous variables, with a higher score indicating more positive perceptions towards the physical environment. An example of a statement for ‘perceived safety’ is: ‘There is not a lot of busy traffic in the neighborhood’, but also: ‘Crime levels are low in the neighbourhood’. In addition, we studied the item on air pollution and the item on litter, rubbish or graffiti as single categorical variables in three categories: agree, neutral and disagree.

### Exposure measure–perceived social environment

The social environment was assessed using a 13-item questionnaire developed by Beenackers *et al*. [45] that was previously validated in this sample [46]. All items were assessed using a 5-point Likert scale ranging from ‘strongly disagree’ to ‘strongly agree’. Based on a reliability analysis we distinguished two domains of neighborhood social capital: social network and social cohesion [46]. To examine these domains in a neighborhood context, we aggregated individual scores at the neighborhood level. The domains were operationalized as continuous variables, with social network ranging from 7.90 to 12.69, and social cohesion ranging from 13.33 to 19.75. Additionally, the single item ‘most people in this neighborhood can be trusted’ was used as an indicator of neighborhood levels of trust and assessed in three categories based on the distribution of the data.

### Covariates and potential moderators

We obtained data from the survey on age, gender, presence of children in the household, level of education, employment status, urban region of residence, years of residency and whether most of the participants’ leisure time was spent in the neighborhood (yes/no). Age (18 to 65, 65+) and years of residency in the neighborhood (0–9, 10+) were dichotomized. Since education systems differed between the participating countries, level of education was also dichotomized (lower: from less than primary school to higher secondary education, higher: college or university level). We divided employment status into three groups: employed/in education, not employed/not in education, and retired.

### Statistical analysis

Participants for whom no address was known (*n* = 162), who lived in a neighborhood that was not covered by GSV at the time of data collection (*n* = 84), or who could not be allocated to one of the remaining 59 neighborhoods (*n* = 586) were excluded from analysis. This left 5,205 participants available for analysis. The analyses were conducted using Stata version 12 [47].

Descriptive characteristics and univariate comparisons of the total sample and by four levels of happiness were assessed using One-way ANOVAs and Chi-Square tests. Comparisons for non-normally distributed continuous variables were assessed using the Kruskal-Wallis One-way ANOVA. We separately examined the relations between the fourteen independent variables and happiness, by conducting multivariable multinomial logistic regression analyses with clustered standard errors at the neighborhood level. By using clustered standard errors, the regression coefficients’ standard errors were corrected for the correlated clusters within neighborhoods. A multinomial logistic regression analysis provides relative risk ratios (RRR), but for the sake of interpretation of the results we refer to the ‘likelihood’ of being happy, rather than to the ‘relative risk’ to be happy. We used the ‘unhappy’ category as a reference group to gain insight in the neighbourhood factors associated with ‘being happier’. Since the objectively assessed domains originally did not contain values >1 (traffic safety: 0.01–0.62, functionality: 0.12–0.69, destinations: 0.01–0.12, aesthetics: 0.19–0.71) we related a 0.1-point (instead of a 1-point) change in these variables to likelihood of happiness.

Missing values ranged from <1% (employment status) to 24.3% (perceived functionality) and were handled using the multiple imputation technique, based on an assumption that values were missing at random. With the multiple imputation technique, missing values are replaced by plausible values multiple times to account for the uncertainty of these values [48]. Given the percentage of missing values, ten complete datasets were generated using predictive mean matching, which were, after analysis, pooled into one final data set [49,50]. As a sensitivity check, all final analyses were repeated using complete cases only.

After multiple imputation, we tested for moderation by age, gender, presence of children in the household, level of education, employment status, urban region of residence and the amount of leisure time spent in the neighborhood in all associations of the independent variables with happiness. Years of residency were additionally tested for moderation in the association of trust, social cohesion and social network with happiness. The tests were performed by adding interaction terms to the models, involving the (categories of) independent variables and the (categories of) moderators. Given the large number of tests, a *p*-value for interaction of <0.05 (instead of *p*<0.1) was considered to be statistically significant. Where there were significant moderating effects, analyses were stratified by the factor in question to interpret the direction of the interactions. Those variables that were not moderators were treated as confounders. Analyses were then *a priori* adjusted for age, gender, presence of children in the household, level of education and employment status, and we additionally tested whether urban region of residence and the amount of leisure time spent in the neighborhood were confounding variables. Number of years of residency was tested as a confounder solely for the social environment-happiness association. To obtain a *p*-for trend we performed linear regression analyses with happiness as a continuous dependent variable, to detect possible dose-response associations.

## Results

Table 1 presents the socio-demographic and socio-economic characteristics of the total sample and the descriptive statistics of the environmental aspects. Table A in S1 File presents the univariate analysis, showing that moderately (42.2% of the sample) and very happy (40.1% of the total sample) individuals more often lived in neighborhoods with higher levels of aesthetics and perceived their neighborhood to be safer, more functional and free from rubbish, litter and graffiti. Furthermore, these univariate analyses showed that they lived in neighborhoods with more social cohesion and were more likely to trust their neighbors.

**Table 1:** Characteristics of the total sample.

Variable	Total sample (N = 5205)
**Age (years)**	52.1 ± 16.3
**Gender (% female)**	55.4
**Children (% household without children)**	70.1
**Education (% higher)**	54.2
**Employment status**	
** - % employed/in education**	58.7
** - % unemployed**	12.2
** - % retired**	29.1
**Urban region of residence**	
** - Ghent region (Belgium)**	32.5
** - Greater Paris (France)**	13.5
** - Greater Budapest (Hungary)**	14.1
** - the Randstad (the Netherlands)**	29.9
** - Greater London (UK)**	9.9
**Spending most spare time in neighborhood (% yes)**	72.4
**Years lived in neighborhood (% ≥10)**	64.5
***Objectively assessed physical environment***	
**Traffic safety (0.01–0.62)**	0.26 ± 0.14
**Functionality (0.12–0.69)**	0.38 ± 0.15
**Destinations (0.01–0.12) (Median, (IQR))**	0.02 (0.02)
**Aesthetics (0.19–0.71)**	0.52 ± 0.13
**Water/green spaces (0.00–1.00)**	0.39 ± 0.34
***Perceived physical environment***	
**Safety (1–5)**	3.18 ± 0.65
**Functionality (1–5)**	3.47 ± 0.74
**Destinations (1–2) (Median, (IQR))**	1.67 (1.33)
**Aesthetics (1–5)**	3.58 ± 0.88
**No air pollution**	
** - % Agree**	34.3
** - % Neutral**	27.3
** - % Disagree**	38.4
**No rubbish/litter/graffiti**	
** - % Agree**	53.6
** - % Neutral**	12.5
** - % Disagree**	33.9
***Social environment***	
**Social network (7.90–12.69)**	10.42 ± 1.18
**Social cohesion (13.33–19.75)**	17.36 ± 1.54
**Trust**	
** - % Agree**	56.6
** - % Neutral**	32.3
** - % Disagree**	11.0

*Note*: **Bold** numbers represent *p*-values below the 0.05 level. Values are percentages or mean ± SD, unless indicated otherwise. N per variable may vary due to missing values. IQR = Interquartile range.

### Objectively assessed physical environment

Associations between aspects of the objectively assessed physical environment and level of happiness based on the multivariable multinomial logistic regression analyses are presented in Table 2. In general, we found negative associations of traffic safety, functionality, and amount of destinations in the neighborhood with happiness, and positive dose-response relations of aesthetics and water and green spaces with levels of happiness. We found evidence that employment status moderated the association of traffic safety and functionality of the neighborhood with happiness, thus we presented these associations stratified by employment status (Table 2). Additionally, gender and urban residence moderated the association of destinations in the neighborhood with happiness, so these analyses were stratified as well. We did not find any statistically significant interactions by age, presence of children in the household, level of education or the amount of leisure time spent in the neighborhood.

**Table 2:** RRR and 95%-CI for the association between objectively assessed physical environmental aspects and happiness as derived from multinomial logistic regression analyses with clustered errors (N = 5205).

Objective physical environmental aspect	Unhappy	Neutral	Moderately happy	Very happy	
	(Ref.)	RRR (95%-CI)	RRR (95%-CI)	RRR (95%-CI)	*p* for trend
	N = 227	N = 694	N = 2193	N = 2091	
**1. Traffic safety**^**a**^^,^^**1**^					
** - Employed**	-	1.06 (0.84, 1.34)	0.96 (0.77, 1.20)	0.92 (0.74, 1.15)	0.092
** - Unemployed**	-	0.97 (0.74, 1.26)	0.86 (0.67, 1.12)	0.72 (0.58, 0.89)*	0.002
** - Retired**	-	0.76 (0.60, 0.97)*	0.74 (0.60, 0.90)*	0.70 (0.55–0.89)*	0.019
**2. Functionality**^**b**^^,^^**1**^					
** - Employed**	-	1.07 (0.84, 1.36)	0.98 (0.80, 1.18)	0.94 (0.77, 1.14)	0.109
** - Unemployed**	-	0.92 (0.65, 1.31)	0.80 (0.59, 1.10)	0.69 (0.55, 0.87)*	0.002
** - Retired**	-	0.75 (0.60, 0.95)*	0.74 (0.58, 0.93)*	0.66 (0.52, 0.84)*	0.002
**3. Destinations**^**c**^^,^^**1**^					
** - Man**	-	0.50 (0.15, 1.68)	0.40 (0.14, 1.19)	0.19 (0.07, 0.49)*	<0.001
** - Woman**	-	0.74 (0.30, 1.81)	0.48 (0.19, 1.20)	0.38 (0.18, 0.84)*	0.004
** - Ghent region (Belgium)**	-	2.33 (1.10, 4.94)*	1.06 (0.56, 2.00)	0.55 (0.25, 1.22)	0.005
** - Greater Paris (France)**^**2**^	-	0.05 (0.00, 1.09)	0.06 (0.00, 1.31)	0.04 (0.00, 0.83)*	0.053
** - Greater Budapest (Hungary)**	-	0.89 (0.15, 5.33)	0.49 (0.09, 2.80)	0.47 (0.10, 2.31)	0.080
** - the Randstad (the Netherlands)**	-	0.48 (0.08, 2.71)	0.94 (0.11, 8.40)	0.24 (0.03, 2.02)	0.080
** - Greater London (UK)**	-	0.66 (0.10, 4.14)	0.27 (0.07, 1.01)	0.29 (0.07, 1.21)	0.127
**4. Aesthetics**^**d**^^,^^**1**^	-	1.11 (0.97, 1.28)	1.19 (1.07, 1.32)*	1.28 (1.16, 1.42)**	<0.001
**5. Water and green spaces**	-	1.48 (0.89, 2.46)	1.57 (1.02, 2.42)*	1.83 (1.15, 2.93)*	0.038

‘Unhappy’ serves as reference category. Models are adjusted for age, gender, children, educational level, employment status and urban region of residence, except when already stratified by one of these variables.

^a^ Percentage of streets with e.g. pedestrian crossings, bicycle lanes and traffic lights.

^b^ Percentage of streets with structured and well maintained street segments, paths and bus stops etc.

^c^ Percentage of streets with community and commercial facilities available.

^d^ Percentage of streets with public parks; trees; good condition residential buildings etc.

^1^ Domain was multiplied tenfold.

^2^ The reference category for Greater Paris (France) consisted of less than 10 people.

* = *p*-value <0.05

** = *p*-value <0.001.

Among retired individuals, a higher level of traffic safety was associated with a lower likelihood of being very happy (RRR = 0.70, 95%-confidence interval (CI) = 0.55; 0.89), with evidence of a dose- response relation (i.e. a 0.10-point increase in traffic safety (range 0.01–0.62) was associated with a likelihood ratio of 0.7 of being very happy relative to being unhappy). For unemployed individuals, a higher level of traffic safety was also associated with a lower likelihood of being very happy, in comparison to being unhappy. Among employed individuals we found no statistically significant differences. Similar patterns were present in the association between functionality of the neighborhood and happiness. Living in a neighborhood with more destinations was associated with a lower likelihood of being very happy for both men and women (RRR for men = 0.19, 95%-CI = 0.07; 0.49, RRR for women = 0.38, 95%-CI = 0.18; 0.84).

Living in a neighborhood with higher levels of aesthetics was associated with a higher likelihood of being moderately happy (RRR = 1.19, 95%-CI = 1.07; 1.32) or very happy (RRR = 1.28, 95%-CI = 1.16; 1.42). Likewise, living in a neighborhood with more water and green spaces was also associated with a higher likelihood of being moderately (RRR = 1.57, 95%-CI = 1.02; 2.42) or very happy (RRR = 1.83, 95%-CI = 1.15; 2.93). Both associations showed evidence of a dose-response relation. These effect sizes suggest that a 0.1-point increase in aesthetics (range 0.19–0.71) was associated with a 1.3 times higher likelihood of being very happy, and a 1-point increase in water and green spaces (range 0.00–1.00) was associated with a 1.8 times higher likelihood ratio of being very happy compared to being unhappy. Sensitivity analyses with a non-imputed data set yielded similar results (see Table B in S1 File).

### Perceived physical environment

Table 3 presents the association between aspects of the perceived physical environment and level of happiness. In general, we found positive associations between the perceived environmental characteristics and happiness, except for perceived presence of key destinations. Age, gender, presence of children in the household, employment status and leisure time spent in the neighborhood were not moderators in these associations. Although we found statistically significant interactions by urban region of residence, stratified analysis of the variables litter, graffiti and rubbish by urban region of residence lacked sufficient statistical power, since too few respondents per category remained. Educational level was also found to moderate the relation between perceived destinations and perceived air pollution with happiness, and was stratified accordingly (Table 3).

**Table 3:** RRR and 95%-CI for the association between perceived physical environmental aspects and happiness as derived from multinomial logistic regression analyses with clustered errors (N = 5205).

Perceived physical environmental aspect	Unhappy	Neutral	Moderately happy	Very happy	
	(Ref.)	RRR (95%-CI)	RRR (95%-CI)	RRR (95%-CI)	*p* for trend
	N = 227	N = 694	N = 2193	N = 2091	
**1. Safety**^**a**^	-	1.41 (1.05, 1.88)*	1.81 (1.44, 2.29)**	2.29 (1.82, 2.88)**	<0.001
**4. Functionality**^**b**^					
** - Ghent region (Belgium)**	-	1.09 (0.72, 1.65)	1.32 (1.04, 1.67)*	1.65 (1.26, 2.16)**	<0.001
** - Greater Paris (France)**^**1**^	-	0.68 (0.20, 2.32)	0.86 (0.27, 2.70)	1.24 (0.39, 3.88)	0.015
** - Greater Budapest (Hungary)**	-	1.57 (0.92, 2.69)	1.67 (1.06, 2.64)*	1.43 (0.87, 2.34)	0.014
** - the Randstad (the Netherlands)**	-	1.93 (1.32, 2.84)*	2.25 (1.63, 3.10)**	2.99 (2.13, 4.19)**	<0.001
** - Greater London (UK)**	-	2.16 (0.88, 5.34)	2.82 (1.30, 6.11)*	3.20 (1.45, 7.09)*	0.006
**3. Destinations**^**c**^					
** - Lower education**	-	0.87 (0.75, 1.02)	0.80 (0.69, 0.93)*	0.89 (0.76, 1.05)	0.825
** - Higher education**	-	1.13 (0.87, 1.46)	1.07 (0.83, 1.37)	1.22 (0.95, 1.57)	0.021
**2. Aesthetics**^**d**^					
** - Ghent region (Belgium)**	-	1.11 (0.79, 1.57)	1.42 (1.08, 1.86)*	1.72 (1.30, 2.27)**	0.005
** - Greater Paris (France)**^**1**^	-	1.29 (0.56, 2.98)	1.70 (0.71, 4.06)	1.96 (0.78, 4.93)	0.053
** - Greater Budapest (Hungary)**	-	1.13 (0.69, 1.86)	1.42 (0.90, 2.26)	1.56 (0.99, 2.46)	0.080
** - the Randstad (the Netherlands)**	-	1.74 (1.20, 2.50)*	1.83 (1.32, 2.54)**	2.88 (2.09, 3.95)**	0.080
** - Greater London (UK)**	-	1.17 (0.74, 1.84)	2.19 (1.43, 3.37)**	2.59 (1.66, 4.05)**	0.127
**5. No air pollution**^**2**^					
** a) Disagree**	-	Ref.	Ref.	Ref.	
** b) Neutral**					
** - Lower education**	-	0.92 (0.54, 1.58)	0.77 (0.45, 1.31)	0.85 (0.50, 1.44)	0.496
** - Higher education**	-	1.13 (0.64, 2.00)	1.20 (0.73, 1.98)	1.36 (0.82, 2.24)	0.123
** c) Agree**					
** - Lower education**	-	0.81 (0.44, 1.47)	1.00 (0.62, 1.63)	1.24 (0.75, 2.05)	0.009
** - Higher education**	-	1.18 (0.60, 2.33)	2.11 (1.17, 3.81)*	2.74 (1.47, 5.11)*	<0.001
**6. No litter/rubbish/graffiti**^**2**^					
** a) Disagree**	-	Ref.	Ref.	Ref.	
** b) Neutral**	-	1.92 (1.24, 2.97)*	1.57 (1.04, 2.39)*	1.63 (1.06, 2.50)*	0.629
** c) Agree**	-	1.41 (0.97, 2.06)	1.97 (1.47, 2.63)**	2.58 (1.88, 3.54)**	<0.001

‘Unhappy’ serves as reference category. Models are adjusted for age, gender, children, educational level and employment status, except when already stratified by one of these variables.

^a^ Perceived safety from crime and safety from traffic.

^b^ Perceived quality and presence of structure in terms of street segments and paths.

^c^ Perceived availability of community and commercial facilities.

^d^ Play areas are well maintained; neighborhood is pleasant to walk/cycle in; neighborhood is free from rubbish/litter/graffiti.

^1^ The reference category for Greater Paris (France) consisted of less than 10 people.

^2^ Additionally adjusted for urban region of residence.

* = *p*-value <0.05

** = *p*-value <0.001.

Individuals who perceived their neighborhood to be safer had a higher likelihood of being happier, with evidence of a dose-response relation. In these associations, a 1-point increase in perceived safety was associated with a 1.8 times higher likelihood of being moderately happy and a 2.3 times higher likelihood of being very happy compared to being unhappy. Among individuals living in Ghent (Belgium), the Randstad (the Netherlands) and greater London (UK), who perceived their neighborhood to be more functional, there was a higher likelihood of being happier, with a dose-response relation. This association was, however, not statistically significant in for moderately happy or very happy individuals living in greater Paris (France), and very happy individuals living in Budapest (Hungary).

Among the Flemish, Dutch and English participants, individuals who perceived their neighborhood to have more favorable aesthetics had a higher likelihood of being very happy (RRR = 1.72 with 95%-CI = 1.26; 2.16, RRR = 2.88 with 95%-CI = 2.13; 4.19, RRR = 2.59 with 95%-CI = 1.45; 7.09, respectively). More highly educated people who perceived the air in their neighborhood not to be polluted also had a higher likelihood of being very happy (RRR = 2.74, 95%-CI = 1.47; 5.11). Furthermore, individuals who perceived that their neighborhood was free from litter, rubbish and graffiti had a higher likelihood of being happier. Sensitivity analyses with a non-imputed data set yielded similar results (see Table C in S1 File).

### Social environment

Social environmental aspects were positively associated with happiness, with evidence of a dose-response effect (Table 4). Gender was a moderator in the association of social network and happiness, but other than that we did not find any statistically significant interactions for the social environmental aspects. Therefore, we stratified social network by gender.

**Table 4:** RRR and 95%-CI for the association between social environmental aspects and happiness as derived from multinomial logistic regression analyses with clustered errors (N = 5205).

Social environmental aspect	Unhappy	Neutral	Moderately happy	Very happy	
	(Ref.)	RRR (95%-CI)	RRR (95%-CI)	RRR (95%-CI)	*p* for trend
	N = 227	N = 694	N = 2193	N = 2091	
**1. Social network**					
** - Male**	-	1.24 (0.97, 1.60)	1.60 (1.25, 2.06)**	1.74 (1.32, 2.29)**	<0.001
** - Female**	-	1.27 (1.01, 1.59)*	1.33 (1.05, 1.69)*	1.49 (1.22, 1.82)**	<0.001
**2. Social cohesion**	-	1.08 (0.98, 1.19)	1.22 (1.10, 1.35)**	1.31 (1.19, 1.44)**	<0.001
**3. Trust**					
** a) Disagree**	-	Ref.	Ref.	Ref.	
** b) Neutral**	-	1.45 (0.97, 2.15)	1.76 (1.19, 2.60)*	2.04 (1.30, 3.19)*	0.005
** c) Agree**	-	1.44 (0.93, 2.21)	2.70 (1.83, 4.00)**	4.33 (2.83, 6.62)**	<0.001

‘Unhappy’ serves as reference category.

Models are adjusted for age, gender, children, educational level, employment status and urban region of residence, except when already stratified by one of these variables.

* = *p*-value <0.05

** = *p*-value <0.001.

For both men and women, we found that living in a neighborhood with a greater social network was associated with a higher likelihood of being very happy for both men and women (RRR = 1.74 with 95%-CI = 1.32; 2.29 for men, RRR = 1.49 with 95%-CI = 1.22; 1.82 for women). Living in a neighborhood with higher levels of social cohesion was associated with a higher likelihood of being happier (moderately happy: RRR = 1.22, 95%-CI = 1.10; 1.35), very happy: RRR = 1.31, 95%-CI = 1.19; 1.44), meaning that a 1-point increase in social cohesion (range 13.33–19.75) was associated with a 1.3 times higher risk of being very happy relative to being unhappy. Furthermore, individuals who trusted their neighbors had a higher likelihood of being moderately happy (RRR = 2.70, 95%-CI = 1.83; 4.00) and very happy (RRR = 4.33, 95%-CI = 2.83; 6.62). Sensitivity analyses with a non-imputed data set yielded similar results (see Table D in S1 File).

## Discussion

We explored several relations between physical and social environmental aspects and happiness in adults across Europe. In general, we found that living in neighborhoods with higher levels of aesthetics, more water and green spaces that were perceived to be safer, more functional and cleaner, with more social contacts, stronger social cohesion and where neighbors are trusted, was associated with higher levels of happiness. Additionally, we found that objective measures of traffic safety, functionality of the neighborhood and presence of key destinations in the neighborhood were inversely associated with happiness. We also found negative associations between the perceived number of these destinations and happiness. Some associations were moderated by socio-demographic or socio-economic factors, but for nearly all associations the direction was similar across men and women, higher and lower educated individuals, employed, unemployed and retired individuals and across European regions in the study.

The positive association between objectively assessed water and green spaces and happiness is consistent with what has been found in previous studies [25,51,52]. One hypothesis to explain this is that of biophilia, which states that humans have an innate tendency to affiliate with other forms of life [53]. Living in a natural neighborhood, e.g., a neighborhood with more water and green spaces, contributes to fulfilling this need, and this is argued to lead to a greater well-being [54]. Another possible explanation for the association of water and green spaces with happiness is that natural environments are associated with lower levels of stress and may have a restorative effect [55]. Furthermore, green environments have been associated with higher engagement in physical activity, and some evidence suggests that green spaces promote social cohesion and interaction [55,56], which are in turn well-established predictors of happiness [9,22,57,58].

In addition to an environment with more water and green space, we found that (perceived and objectively assessed) level of aesthetics in the neighborhood was associated with happiness. This is in line with the findings of Leyden *et al*., who concluded that aesthetics of the neighborhood matter for individual happiness [15]. Indeed, our findings showed that individuals who reported that their neighborhood was free from rubbish, litter and graffiti, and thus generally perceived as visually more pleasant, were happier. Furthermore, we found that individuals living in a neighborhood that they perceived to be more functional (choice of routes, usually slow traffic, and sufficient pedestrian crossings to cross busy roads) were happier. This finding might be explained by a neighborhood that is more functional being more conducive to social interactions, since it reduces barriers to social connections, and greater happiness is associated with some forms of social capital [59]. In all of these cases reversed causality is also possible, whereby happiness affects the choice of a place to live. For instance, happy people could give more priority to aesthetic issues and closeness to water and green spaces, both because of effects of happiness itself or as a result of tendencies related to happiness [60], such as more frequent sporting and lower materialism [61].

Furthermore, this study provided further evidence of the strong association of social networks, social cohesion and trust with happiness. We found that these associations were similar among indicators of socio-economic and socio-demographic groups, and across urban European regions of the SPOTLIGHT study. Previous studies have consistently found a link between social capital and happiness [9,22,58]. Rodríguez-Pose and von Berlepsch also found that social interaction and trust were associated with happiness in a large cohort in several European countries [16]. Leyden *et al*. found that social cohesion in the neighborhood is positively associated with happiness in ten major world cities [15]. It is argued that this strong association exists because people with close friends, neighbors and spouses are less likely to express a range of characteristics associated with diminished subjective well-being, such as sadness, loneliness and low self-esteem [58]. It is yet to be determined whether all these social environmental factors are a proxy for the broader social environment, or whether specific social factors such as perceived social support are important for happiness. Reversed causality can also be involved, since it has been found that happy people are also tend to more easily built social networks [62].

There were differences in associations observed with *objectively* and *subjectively* measured safety and functionality. Previous studies, including a study using the same dataset as this study, have also found differences between objective and subjective measures of the environment [63,64]. The objectively verifiable presence of certain environmental characteristics in a neighborhood increases the probability that an individual will be exposed to them, but this is not a certainty. The strength of the association may depend on the level of ‘conscious’ exposure and may be influenced by personality, experiences and cognitive capacities [30]. Some environments may be objectively functional for some individuals, but less so for other individuals who have different requirements of their environment. Alternatively, some objectively assessed ‘safe’ neighborhoods may actually be perceived as unsafe.

We found negative associations between objectively assessed traffic safety and functionality and happiness. Although this may seem counterintuitive, it could be that measures to improve traffic safety and functionality have been put in place due to traffic unsafety (e.g. because of heavy traffic), like pedestrian crossings, bicycle lanes and traffic lights. As such, these measures may have made the neighborhood safer or more pleasant for walking and cycling [65], but residents may still be aware of the dangers or unpleasantness that required these measures and, as such, result in lower levels of happiness. Indeed, heavy traffic is generally associated with more traffic nuisance and air pollution, which is in turn negatively related to well-being [28,66,67]. This corresponds with our finding that individuals who lived in neighborhoods where the air was not perceived to be polluted were happier, though this relation was only statistically significant among the higher educated.

We found some inverse–but mostly non-significant- associations for the objective and perceived assessed destinations with happiness. These negative associations can possibly be explained by the fact that neighborhoods with more destinations were likely to be denser, with a more urbanized built environment, and less of a natural environment. However, this is in contrast with the study of Leyden *et al*., who found that easy access to culture and leisure community facilities was positively associated with happiness [15].

### Strengths and limitations

Our study has several strengths. We made use of a dataset from a large survey that was conducted using consistent methods across five European urban regions, which increased its generalisability. This study was the first to combine factors of different contextual domains in relation with happiness, with several physical as well as social environmental factors included. Furthermore, our study contributed to understanding the associations between perceived and objective measures of the physical environment, and how they vary within different subgroups of the population. The objective measures of the environment were obtained using an innovative and validated tool [39].

There are also some limitations to our study. Firstly, this was a cross-sectional study, thus causality cannot be determined. Secondly, there was a low response rate (10.8%). Although this is not unusual in studies on this scale, this could have led to selection bias. Third, although the question we used to assess happiness is an accepted question in research on this topic, there is no gold standard measure of this construct, and self-reported measures of happiness can also be vulnerable to a variety of response biases [68,69]. The questionnaire was provided in multiple languages according to the participants’ residential language, but even though a back and forward translation technique was applied, the construct of ‘happiness’ may have a slightly different meaning in each of the five participating urban regions. As a result, this question may have been interpreted slightly different by respondents in different countries [68]. However, research on happiness in multi-lingual Switzerland did not find this an issue between French, German and Italian [70]. Fourth, associations between physical environmental perceptions and happiness may be spurious, because participants’ happiness can influence their environmental perceptions [71]. Finally, the use of regression analyses with clustered standard errors at the neighbourhood level may have resulted in wider confidence intervals, but our software did not allow for a multilevel analysis while using multiple imputed data and a categorical outcome variable.

## Conclusions

In conclusion, we found that several aspects of the social and physical neighborhood were associated with levels of happiness in an urban European population, across different socio-economic and socio-demographic subgroups. Future studies could investigate whether the association of happiness with the functionality of the neighborhood and a green environment with happiness is mediated by social capital or physical activity. If future studies are able to confirm causality in these associations using a longitudinal or “natural experimental” design—e.g., by following people who move to different neighborhoods—the arguments for using urban design to promote happiness, and with it, presumably, the overall health of the general population, will be strengthened.

## Supporting information

S1 File Supplementary tables. (DOCX)
