# Supplementary material for: Contextual correlates of happiness in European adults
Source: PLoS One. 2018 Jan 24;13(1):e0190387. doi: 10.1371/journal.pone.0190387 (PMC5783333; doi:10.1371/journal.pone.0190387)
Supplement: S1 File — (DOCX) [file pone.0190387.s001.docx]

**Supplemental material – Contextual correlates of happiness in European adults**

E.A.C. Hart, J. Lakerveld, M. McKee, J.M. Oppert, H. Rutter, H. Charreire, R. Veenhoven, H. Bárdos, S. Compernolle, I. De Bourdeaudhuij, J. Brug, J.D. Mackenbach.

Table of contents

**[Table A](#_Toc502219545)** [2](#_Toc502219545)

[**Table B** 3](#_Toc502219546)

[**Table C** 4](#_Toc502219547)

[**Table D** 5](#_Toc502219548)

**Table A.** Characteristics and univariate comparisons by level of happiness.

| **Variable** | **Unhappy**  **(N= 208)** | **Neutral**  **(N= 639)** | **Moderately happy**  **(N= 2023)** | **Very happy**  **(N= 1925)** | **F/**  **Chi^2^** |
| --- | --- | --- | --- | --- | --- |
| **Age (years)** | 52.2 ± 16.9 | 55.5 ± 16.1 | 50.0 ± 16.0 | 53.2 ± 16.4 | **23.51** |
| **Gender (% female)** | 53.4 | 55.2 | 57.2 | 53.8 | 4.74 |
| **Children (% household without children)** | 74.0 | 76.4 | 68.8 | 69.1 | **15.84** |
| **Education (% higher)** | 47.0 | 37.3 | 58.6 | 55.9 | **92.86** |
| **Employment status** |  |  |  |  | **113.66** |
| - **% employed/in education** | 49.0 | 46.6 | 64.3 | 57.8 |  |
| - **% unemployed** | 25.5 | 16.2 | 11.5 | 10.1 |  |
| - **% retired** | 25.5 | 37.2 | 24.2 | 32.0 |  |
| **Urban region of residence** |  |  |  |  | **456.60** |
| - **Ghent region (Belgium)** | 41.3 | 40.4 | 24.8 | 37.1 |  |
| - **Greater Paris (France)^2^** | 2.4 | 14.4 | 17.3 | 10.4 |  |
| - **Greater Budapest (Hungary)** | 12.5 | 13.5 | 21.4 | 7.0 |  |
| - **the Randstad (the Netherlands)** | 32.7 | 27.7 | 21.8 | 38.9 |  |
| - **Greater London (UK)** | 11.1 | 4.1 | 14.8 | 6.6 |  |
| **Spending most spare time in neighborhood (% yes)** | 74.3 | 74.7 | 71.7 | 72.1 | 2.52 |
| **Years lived in neighborhood (% ≥10)** | 59.2 | 68.0 | 62.9 | 65.7 | **9.43** |
| ***Objectively assessed physical environment*** |  |  |  |  |  |
| **Traffic safety (0.01-0.62)** | 0.26 ± 0.14 | 0.27 ± 0.14 | 0.26 ± 0.15 | 0.27 ± 0.13 | 1.91 |
| **Functionality (0.12-0.69)** | 0.39 ± 0.16 | 0.37 ± 0.15 | 0.38 ± 0.15 | 0.39 ± 0.16 | 1.18 |
| **Destinations (0.01-0.12) (Median, (IQR))** | 0.031 (0.03) | 0.030 (0.03) | 0.035 (0.03) | 0.026 (0.02) | **79.61** |
| **Aesthetics (0.19-0.71)** | 0.51 ± 0.12 | 0.51 ± 0.13 | 0.50 ± 0.12 | 0.54 ± 0.12 | **40.63** |
| **Water/green spaces (0.00-1.00)** | 0.39 ± 0.33 | 0.39 ± 0.35 | 0.33 ± 0.33 | 0.46 ± 0.35 | **43.12** |
| ***Perceived physical environment*** |  |  |  |  |  |
| **Safety (1-5)** | 2.89 ± 0.67 | 3.04 ± 0.65 | 3.18 ± 0.65 | 3.27 ± 0.65 | **30.02** |
| **Functionality (1-5)** | 3.12 ± 0.73 | 3.35 ± 0.74 | 3.44 ± 0.73 | 3.58 ± 0.73 | **29.15** |
| **Destinations (1-2) (Median, (IQR))** | 1.67 (1.33) | 1.67 (1.33) | 1.00 (0.67) | 1.67 (1.33) | **16.67** |
| **Aesthetics (1-5)** | 3.13 ± 0.89 | 3.32 ± 0.87 | 3.55 ± 0.87 | 3.75 ± 0.87 | **54.65** |
| **No air pollution** |  |  |  |  | **55.80** |
| - **% Agree** | 39.7 | 39.2 | 35.8 | 30.5 |  |
| - **% Neutral** | 31.2 | 32.6 | 26.1 | 26.4 |  |
| - **% Disagree** | 29.1 | 28.2 | 38.1 | 43.1 |  |
| **No rubbish/litter/graffiti** |  |  |  |  | **112.61** |
| - **% Agree** | 39.4 | 41.7 | 51.6 | 61.2 |  |
| - **% Neutral** | 12.8 | 18.2 | 11.7 | 11.4 |  |
| - **% Disagree** | 47.8 | 40.1 | 36.7 | 27.4 |  |
| ***Social environment*** |  |  |  |  |  |
| **Social network (7.90-12.69)** | 10.16 ± 1.19 | 10.36 ± 1.13 | 10.19 ± 1.21 | 10.72 ± 1.08 | **77.21** |
| **Social cohesion (13.33-19.75)** | 17.02 ± 1.64 | 17.09 ± 1.67 | 17.18 ± 1.48 | 17.69 ± 1.50 | **49.61** |
| **Trust** |  |  |  |  | **180.79** |
| - **% Disagree** | 20.8 | 17.1 | 12.0 | 7.0 |  |
| - **% Neutral** | 36.6 | 39.9 | 35.5 | 26.0 |  |
| - **% Agree** | 42.6 | 43.0 | 52.4 | 67.0 |  |

**Table B.** RRR and 95%-CI for the association between the objectively assessed physical environmental aspects and happiness as derived from multinomial logistic regression analyses with clustered errors (complete case analysis)

| **Objective physical environment** | **Unhappy** | **Neutral** | **Moderately happy** | **Very happy** |  |
| --- | --- | --- | --- | --- | --- |
|  | **(Ref.)** | **RRR (95%-CI)** | **RRR (95%-CI)** | **RRR (95%-CI)** | ***p* for trend** |
| 1. **Traffic safety^1, 5^** |  |  |  |  |  |
| - **Employed** | - | 1.16 (0.91, 1.47) | 1.01 (0.79, 1.29) | 0.96 (0.75, 1.22) | 0.064 |
| - **Unemployed** | - | 1.01 (0.77, 1.31) | 0.83 (0.64, 1.09) | 0.70 (0.57, 0.86)* | <0.001 |
| - **Retired** | - | 0.73 (0.56, 0.94)* | 0.69 (0.54, 0.86)* | 0.65 (0.50, 0.83)* | 0.002 |
|  |  |  |  |  |  |
| **2. Functionality^2, 5^** |  |  |  |  |  |
| - **Employed** | - | 1.13 (0.88, 1.44) | 1.01 (0.82, 1.24) | 0.96 (0.78, 1.18) | 0.083 |
| - **Unemployed** | - | 0.98 (0.70, 1.37) | 0.79 (0.58, 1.09) | 0.68 (0.53, 0.87)* | 0.001 |
| - **Retired** | - | 0.71 (0.56, 0.89)* | 0.69 (0.54, 0.88)* | 0.61 (0.48, 0.77)** | <0.001 |
|  |  |  |  |  |  |
| **3. Destinations^3, 5^** |  |  |  |  |  |
| - **Male** | - | 0.59 (0.13, 2.65) | 0.40 (0.11, 1.48) | 0.17 (0.05, 0.53)* | <0.001 |
| - **Female** | - | 0.71 (0.29, 1.71) | 0.34 (0.14, 0.84)* | 0.31 (0.15, 0.64)* | 0.001 |
|  |  |  |  |  |  |
| - **Ghent region (Belgium)** | - | 4.64 (2.15, 10.00)** | 1.72 (0.95, 3.09) | 0.79 (0.36, 1.72) | <0.001 |
| - **Greater Paris (France)^6^** | - | - | - | - |  |
| - **Greater Budapest (Hungary)** | - | 0.82 (0.13, 5.09) | 0.37 (0.06, 2.35) | 0.43 (0.09, 2.13) | 0.032 |
| - **the Randstad (the Netherlands)** | - | 0.31 (0.07, 1.28) | 0.89 (0.11, 7.27) | 0.16 (0.02, 1.47) | 0.067 |
| - **Greater London (UK)** | - | 1.46 (0.22, 9.77) | 0.21 (0.06, 0.78)* | 0.26 (0.05, 1.30) | 0.102 |
|  |  |  |  |  |  |
| **2. Aesthetics^4, 5^** | - | 1.10 (0.94, 1.28) | 1.21 (1.08, 1.35)* | 1.30 (1.18, 1.43)** | <0.001 |
|  |  |  |  |  |  |
| **5. Water and green spaces** | - | 1.45 (0.86, 2.45) | 1.58 (1.05, 2.37)* | 1.83 (1.15, 2.91)* | 0.034 |

With ‘unhappy’ serving as reference category. N per variable may vary due to missing values. Models are adjusted for age, gender, children, educational level, employment status and urban region of residence, except when already stratified by one of these variables.
^1^ Percentage of streets with e.g. pedestrian crossings, bicycle lanes and traffic lights. ^2^ Percentage of streets with structured and well maintained street segments, paths and bus stops etc. ^3^ Percentage of streets with community and commercial facilities available. ^4^ Percentage of streets with public parks; trees; good condition residential buildings etc.
^5^ Domain is multiplied tenfold. ^6^ No valid estimates available due to power restrictions.
*= *p*-value <0.05, **= *p*-value <0.001.

**Table C.** RRR and 95%-CI for the association between perceived physical environmental aspects and happiness as derived from multinomial logistic regression analyses with clustered errors (complete case analysis)

| **Perceived physical environment** | **Unhappy** | **Neutral** | **Moderately happy** | **Very happy** |  |
| --- | --- | --- | --- | --- | --- |
|  | **(Ref.)** | **RRR (95%-CI)** | **RRR (95%-CI)** | **RRR (95%-CI)** | ***p* for trend** |
| **1. Safety^1^** | - | 1.48 (1.10, 2.00)* | 1.95 (1.52, 2.51)** | 2.43 (1.91, 3.08)** | <0.001 |
| **4. Functionality^2^** |  |  |  |  |  |
| - **Ghent region (Belgium)** | - | 1.24 (0.72, 2.15) | 1.42 (1.04, 1.96)* | 1.90 (1.32, 2.73)* | <0.001 |
| - **Greater Paris (France)^5^** | - | - | - | - |  |
| - **Greater Budapest (Hungary)** | - | 1.98 (1.01, 3.90)* | 1.72 (1.04, 2.85)* | 1.36 (0.79, 2.32) | 0.236 |
| - **the Randstad (the Netherlands)** | - | 2.15 (1.49, 3.11)** | 2.62 (1.91, 3.61)** | 3.49 (2.74, 4.44)** | <0.001 |
| - **Greater London (UK)** | - | 1.64 (0.58, 4.65) | 2.57 (1.09, 6.04)* | 2.97 (1.18, 7.47) | 0.046 |
| **3. Destinations^3^** |  |  |  |  |  |
| - **Lower education** | - | 0.86 (0.75, 1.00)* | 0.77 (0.65, 0.92)* | 0.87 (0.72, 1.05) | 0.745 |
| - **Higher education** | - | 1.18 (0.94, 1.46) | 1.08 (0.87, 1.34) | 1.25 (1.01, 1.55)* | 0.014 |
| **2. Aesthetics^4^** |  |  |  |  |  |
| - **Ghent region (Belgium)** | - | 1.25 (0.88, 1.78) | 1.57 (1.18, 2.08)* | 1.94 (1.50, 2.51)** | <0.001 |
| - **Greater Paris (France)^5^** | - | - | - | - |  |
| - **Greater Budapest (Hungary)** | - | 1.23 (0.72, 2.09) | 1.43 (0.85, 2.39) | 1.58 (0.93, 2.67) | 0.030 |
| - **the Randstad (the Netherlands)** | - | 1.67 (1.23, 2.27)* | 1.80 (1.37, 2.37)** | 2.83 (2.16, 3.70)** | <0.001 |
| - **Greater London (UK)** | - | 1.29 (0.84, 1.98) | 2.39 (1.57, 2.65)** | 2.86 (1.73, 4.73)** | 0.015 |
| **5. No air pollution^6^** |  |  |  |  |  |
| 1. **Disagree** | - | Ref. | Ref. | Ref. |  |
| 1. **Neutral** |  |  |  |  |  |
| - **Lower education** | - | 0.82 (0.49, 1.40) | 0.69 (0.41, 1.17) | 0.74 (0.43, 1.25) | 0.289 |
| **- Higher education** | - | 1.13 (0.65, 1.96) | 1.28 (0.76, 2.16) | 1.44 (0.84, 2.45) | 0.075 |
| 1. **Agree** |  |  |  |  |  |
| **- Lower education** | - | 0.74 (0.43, 1.28) | 1.03 (0.66, 1.60) | 1.20 (0.74, 1.95) | 0.007 |
| **- Higher education** | - | 1.26 (0.62, 2.55) | 2.57 (1.39, 4.72)* | 3.24 (1.70, 6.18)** | <0.001 |
| **6. No litter/rubbish/graffiti^6^** |  |  |  |  |  |
| 1. **Disagree** | - | Ref. | Ref. | Ref. |  |
| 1. **Neutral** | - | 1.84 (1.17, 2.90)* | 1.45 (0.95, 2.22) | 1.53 (0.99, 2.37) | 0.838 |
| 1. **Agree** | - | 1.44 (0.95, 2.19) | 1.98 (1.42, 2.75)** | 2.54 (1.81, 3.56)** | <0.001 |

With ‘unhappy’ serving as reference category. N per variable may vary due to missing values. Models are adjusted for age, gender, children, educational level and employment status, except when already stratified by one of these variables. ^1^ Perceived safety from crime and safety from traffic. ^2^ Perceived quality and presence of structure in terms of street segments and paths. ^3^Perceived availability of community and commercial facilities. ^4^ Play areas are well maintained; neighborhood is pleasant to walk/cycle in; neighborhood is free from rubbish/litter/graffiti.
^5^ No valid estimates available due to power restrictions. ^6^ Additionally adjusted for urban region of residence.
*= *p*-value <0.05, **= *p*-value <0.001.

**Table D.** RRR and 95%-CI for the association between social environmental aspects and happiness as derived from multinomial logistic regression analyses with clustered errors (complete case analysis)

| **Social environment** | **Unhappy** | **Neutral** | **Moderately happy** | **Very happy** |  |
| --- | --- | --- | --- | --- | --- |
|  | **(Ref.)** | **RRR (95%-CI)** | **RRR (95%-CI)** | **RRR (95%-CI)** | ***p* for trend** |
| **1. Social network** |  |  |  |  |  |
| - **Male** | - | 2.27 (1.01, 1.59)* | 1.62 (1.29, 2.05)** | 1.74 (1.33, 2.28)** | <0.001 |
| - **Female** | - | 1.32 (1.09, 1.61)* | 1.38 (1.12, 1.71)* | 1.53 (1.29, 1.82)** | <0.001 |
|  |  |  |  |  |  |
| **2. Social cohesion** | - | 1.08 (0.99, 1.19) | 1.23 (1.12, 1.35)** | 1.31 (1.21, 1.43)** | <0.001 |
|  |  |  |  |  |  |
| **3. Trust** |  |  |  |  |  |
| 1. **Disagree** | - | Ref. | Ref. | Ref. |  |
| 1. **Neutral** | - | 1.49 (1.00, 2.22)* | 1.78 (1.20, 2.65)* | 2.08 (1.32, 3.28)* | 0.008 |
| 1. **Agree** | - | 1.48 (0.94, 2.33) | 2.90 (1.93, 4.35)** | 4.60 (2.86, 7.42)** | <0.001 |

With ‘unhappy’ serving as reference category. N per variable may vary due to missing values.
Models are adjusted for age, gender, children, educational level, employment status and urban region of residence, except when already stratified by one of these variables.
*= *p*-value <0.05, **= *p*-value <0.001.
